# Dopamine D1/D5, But not D2/D3, Receptor Dependency of Synaptic Plasticity at Hippocampal Mossy Fiber Synapses that Is Enabled by Patterned Afferent Stimulation, or Spatial Learning

**DOI:** 10.3389/fnsyn.2016.00031

**Published:** 2016-09-23

**Authors:** Hardy Hagena, Denise Manahan-Vaughan

**Affiliations:** Department of Neurophysiology, Medical Faculty, Ruhr University BochumBochum, Germany

**Keywords:** CA3, mossy fibers, dopamine, D1/D5, D2/D3, learning, synaptic plasticity, *in vivo*

## Abstract

Although the mossy fiber (MF) synapses of the hippocampal CA3 region display quite distinct properties in terms of the molecular mechanisms that underlie synaptic plasticity, they nonetheless exhibit persistent (>24 h) synaptic plasticity that is akin to that observed at the Schaffer collateral (SCH)-CA1 and perforant path (PP)-dentate gyrus (DG) synapses of freely behaving rats. In addition, they also respond to novel spatial learning with very enduring forms of long-term potentiation (LTP) and long-term depression (LTD). These latter forms of synaptic plasticity are directly related to the learning behavior: novel exploration of generalized changes in space facilitates the expression of LTP at MF-CA3 synapses, whereas exploration of novel configurations of large environmental features facilitates the expression of LTD. In the absence of spatial novelty, synaptic plasticity is not expressed. Motivation is a potent determinant of whether learning about the spatial experience effectively occurs and the neuromodulator dopamine (DA) plays a key role in motivation-based learning. Prior research on the regulation by DA receptors of long-term synaptic plasticity in CA1 and DG synapses *in vivo* suggests that whereas D2/D3 receptors may modulate a general predisposition toward expressing plasticity, D1/D5 receptors may directly regulate the direction of change in synaptic strength that occurs during learning. Although the CA3 region is believed to play a pivotal role in many forms of learning, the role of dopamine receptors in persistent (>24 h) forms of synaptic plasticity at MF-CA3 synapses is unknown. Here, we report that whereas pharmacological antagonism of D2/D3 receptors had no impact on LTP or LTD, antagonism of D1/D5 receptors significantly impaired LTP and LTD that were induced by solely by means of patterned afferent stimulation, or LTP/LTD that are typically enhanced by the conjunction of afferent stimulation and novel spatial learning. These data indicate an important role for DA acting on D1/D5 receptors in the support of long-lasting and learning-related forms of synaptic plasticity at MF-CA3 synapses and provide further evidence for an important neuromodulatory role for this receptor in experience-dependent synaptic encoding in the hippocampal subfields.

## Introduction

Synaptic plasticity in the hippocampus, in the form of long-term potentiation (LTP) and long-term depression (LTD), comprises the mechanistic foundation for learning and memory processes. As experience-dependent phenomena, both LTP and LTD are strongly influenced by behavioral state and the neuromodulators that mediate state-dependency. In this regard, the neurotransmitter dopamine (DA) stands out as a neuromodulator that is crucial for the fine-tuning of multiple hippocampal functions such as memory acquisition (O’Carroll et al., 2006; Bethus et al., 2010; Heath et al., 2015) and consolidation (Sara et al., 1999; Atherton et al., 2015), as well as in learning related to fear-conditioning (Inoue et al., 2000; Wen et al., 2014; Menezes et al., 2015). DA (in higher concentrations) influences the interaction of synapses with each other, thereby supporting persistent memory storage (Shetty et al., 2015). Furthermore, very long lasting forms of LTP and LTD are believed to comprise cellular correlates for long-term and persistent memory (Lynch, 2004; Malenka and Bear, 2004; Kemp and Manahan-Vaughan, 2007), and several studies report that the dopaminergic system, and in particular the D1/D5 receptors are important for the longevity of synaptic plasticity (Frey et al., 1990, 1991, 1993; Kulla and Manahan-Vaughan, 2000; Lemon and Manahan-Vaughan, 2006; Wiescholleck and Manahan-Vaughan, 2014).

DA receptors can be divided into two groups, comprising the D1-like receptors, that include D1 and D5 receptors, which are positively coupled to adenylyl-cyclase (AC), and the D2-like receptors that include the D2, D3 and D4 subtypes of DA receptors and which are negatively coupled to AC (Andersen et al., 1990; Niznik and Van Tol, 1992; Vallone et al., 2000; Beaulieu and Gainetdinov, 2011). D1/D5-receptors are highly expressed in area CA3 of the hippocampus (Ariano et al., 1997; Ciliax et al., 2000; Khan et al., 2000), whereas D2-like receptor expression in area CA3 seems to be relatively sparse (Goldsmith and Joyce, 1994; Gangarossa et al., 2012).

The main dopaminergic input to the hippocampus stems from the ventral tegmental area (VTA) and is believed to play an important role in supporting the storage of hippocampus-dependent episodic memory (Lisman and Grace, 2005). The VTA is postulated to form a loop with the hippocampus, in which the hippocampus controls the activity of the VTA (Lisman and Grace, 2005) and in turn, the VTA mediates hippocampal activity through the release of DA (Fields et al., 2007). The dopaminergic system is intrinsically involved in different forms of hippocampal synaptic plasticity (Huang and Kandel, 1995; Jay, 2003; Lisman and Grace, 2005; Lemon and Manahan-Vaughan, 2006, 2012; Bethus et al., 2010; Lisman et al., 2011). Pharmacological activation of D1/D5 receptors increases the magnitude and duration of LTP at CA1 and dentate gyrus (DG) synapses of the dorsal hippocampus both *in vivo* and *in vitro* (Otmakhova and Lisman, 1996; Kulla and Manahan-Vaughan, 2000; Li et al., 2003; Lemon and Manahan-Vaughan, 2006; Hamilton et al., 2010; Yang and Dani, 2014). The contribution of D2-like receptors to synaptic plasticity seems to be indirect: activation of these receptors depresses basal synaptic transmission and promotes depotentiation (Manahan-Vaughan and Kulla, 2003). Other studies showed that D2 receptor antagonism modulates effects on spatial recognition memory induced by cholecystokinin B (CCK-B) receptor agonists (Léna et al., 2001). D2-receptor activation in the ventral hippocampus elicits a positive effect on working memory in the form of improved choice accuracy in the radial maze (Wilkerson and Levin, 1999; Rocchetti et al., 2015).

The abovementioned studies, that addressed the role of DA receptors in hippocampal plasticity, have focused exclusively on the CA1 region and the DG. Very little is known about the role of these receptors in synaptic plasticity in the CA3 region, despite its undisputed role in hippocampal information processing and memory (Rolls, 2013; Kesner and Rolls, 2015; Kinnavane et al., 2015). The CA3 region receives information from various inputs within the hippocampus, such as the associational and commissural fibers that arise from CA3 pyramidal cells of the ipsilateral and contralateral hemispheres, respectively, as well as from mossy fibers (MF) and from the perforant path (PP; Blackstad, 1956; Blackstad et al., 1970; Amaral and Dent, 1981; Amaral et al., 1990). MF-CA3 synapses display some unique properties, such as frequency-facilitation (Salin et al., 1996) and presynaptic induction mechanisms for synaptic plasticity (Nicoll and Schmitz, 2005). D1/D5 receptors are expressed in the CA3 region (Ariano et al., 1997; Ciliax et al., 2000; Khan et al., 2000), suggesting that activation of these receptors may modulate synaptic responses at MF-CA3 synapses. In line with this, direct application of DA induces robust potentiation in MF-CA3 synapses of mouse hippocampal slices (Kobayashi and Suzuki, 2007). Furthermore, inhibition of D1/D5 receptors in the CA3 region reduces freezing behavior in a fear conditioning paradigm, equivalent to an impairment of the consolidation phase of fear memory (Wen et al., 2014). This suggests that DA can directly influence information processing at MF-CA3 synapses. However, the role of DA receptors in the neuromodulation of persistent (>24 h) forms of synaptic plasticity at MF-CA3 synapses, that are explicitly associated with hippocampus-dependent memory (Hagena and Manahan-Vaughan, 2011, 2012) has not been studied, so far.

Although traditionally, hippocampal synaptic plasticity is examined using experimental procedures that involve patterned stimulation of afferent fibers, more recently it has become apparent that persistent forms of LTP and LTD can be elicited when weak afferent stimulation (that is insufficient in its own right for the induction of lasting plasticity) is coupled with a novel spatial learning event (Kemp and Manahan-Vaughan, 2007). In behaving rats, coupling a substantial change to the spatial environment with weak afferent stimulation results in input-specific LTP (Kemp and Manahan-Vaughan, 2007). This is a property exhibited by synapses of the CA1 region (Kemp and Manahan-Vaughan, 2004, 2008), the DG (Kemp and Manahan-Vaughan, 2008), and also by commissural/associational-CA3 and MF-CA3 synapses (Hagena and Manahan-Vaughan, 2011). By contrast, robust LTD is elicited when object-place features are added to, or changed within a spatial environment (Kemp and Manahan-Vaughan, 2007). The CA3 region exhibits very striking properties in this regard: discrete object-place constellations facilitate the expression of LTD at commissural/associational-CA3 synapses, whereas large landmark-like object-place constellations facilitate the expression of LTD at MF-CA3 synapses (Hagena and Manahan-Vaughan, 2011). In the CA1 and DG subfields, this kind of learning-facilitated synaptic plasticity is highly sensitive to regulation by the dopaminergic system (Lemon and Manahan-Vaughan, 2006; Wiescholleck and Manahan-Vaughan, 2014), but to what extent learning-facilitated synaptic plasticity at MF-CA3 is subject to dopaminergic modulation is not yet known.

Here, we examined the role of the D1/D5-receptors in both persistent (>24 h) LTP and LTD that were induced by patterned stimulation of MF inputs to the CA3 region of the dorsal hippocampus. In addition, we explored whether these receptors are required for synaptic plasticity at MF synapses that is enhanced by novel spatial learning. We observed that LTP and LTD that is elicited solely by patterned stimulation of afferent fibers is prevented by antagonism of the D1/D5 receptor, but not by antagonism of D2/D3-receptors. Furthermore, D1/D5 antagonism also impaired learning-facilitated plasticity. These results suggest that D1/D5 receptors are crucially involved in modulating synaptic plasticity at MF-CA3 synapses, and specifically in modulating forms that are candidate processes for long-term memory.

## Materials and Methods

The present study was carried out in accordance with the European Communities Council Directive of 22 September 2010 (2010/63/EU) for the care of laboratory animals and after approval of the local government ethics committee (Landesamt für Naturschutz, Umweltschutz und Verbraucherschutz, Nordrhein Westfalen). All efforts were made to minimize the number of animals used.

### Electrophysiology

Seven- to eight-week old male Wistar rats (Charles River, Germany) were anesthetized using sodium pentobarbital, (52 mg/kg, intraperitoneally) and underwent implantation of hippocampal electrodes and a guide cannula, as described previously (Hagena and Manahan-Vaughan, 2010). The recording electrode was placed above the CA3 pyramidal layer of the dorsal hippocampus, 2.9 mm posterior to bregma and 3.0 mm lateral to the midline. A bipolar stimulation electrode was implanted in the mossy fibers, 3.5 mm posterior to bregma and 2.0 mm lateral to the midline. A guide cannula was implanted in the ipsilateral hemisphere to enable injections into the intracerebral ventricle (ICV; Manahan-Vaughan, 1997).

Experiments were commenced 7–10 days after surgery. During all experiments, the animals moved freely in the recording chamber (40 × 40 × 40 cm) and had free access to food and water. To allow the animals to acclimatize they were transferred to the experiment room the day before the experiment took place. Electrophysiological and pharmacological verifications of the validity of the MF-CA3 recordings were conducted during the electrophysiological experiments, and post-mortem verification of electrode sites was conducted, all as described previously (Hagena and Manahan-Vaughan, 2010).

Evoked potentials were analyzed and stored on computer, and the electroencephalogram (EEG) was monitored throughout the experiments. To evoke field excitatory postsynaptic potentials (fEPSPs), a biphasic pulse was given (half-wave duration of 0.2 ms). An input-output curve (stimulation intensity in steps of max. 100 μA in the range of 0–900 μA) was determined immediately prior to commencing the experiment. Test-pulse stimulation in all experiments was conducted using the stimulation intensity that produced an fEPSP that was 40% of the maximum fEPSP obtained in the input-output curve assessment.

To verify stability of recordings, all animals were first tested in a “baseline” experiment where test-pulse stimulation was applied over the same time-period as subsequent plasticity experiments. To induce LTD or STD, low frequency stimulation (LFS) or weak low frequency stimulation (wLFS) consisting of 900 pulses at 1 Hz or 600 pulses at 1 Hz, respectively, were given with a stimulus intensity that yielded potentials that were 70% of the maximum fEPSP observed during the input-output curve analysis. LTP or short-term potentiation (STP) was induced either by high-frequency stimulation (HFS) or weak high-frequency stimulation (wHFS) of afferent fibers. This comprised of four bursts (for LTP) or two bursts (for STP) of 100 pulses at 100 Hz, with a 5 min interburst interval. Animals served as their own controls. Twelve animals were used in this study.

### Spatial Exploration

For the behavioral learning paradigms, animals explored a holeboard (39.8 × 39.8 cm) that was inserted into the recording chamber immediately after the first hour of fEPSP recordings and was left there during wHFS. The holeboard contained four holes (5.5 cm in diameter and 5 cm depth) in each corner. For wLFS experiments, three large objects (5–10 cm in diameter and 5–12 cm in height) were inserted directly into the recording chamber for the duration of wLFS. To ensure maximal familiarization with the recording chamber and external environment, the individual rats were assigned a particular recording chamber where all experiments with the animal were carried out. Re-exposure to the holeboard, or to the large objects (landmarks), took place 1 week after first exposure. The second re-exposure to the holeboard/objects took place a further week after first re-exposure. During re-exposure wHFS experiments the same holeboard was used, and in the case of wLFS experiments, the landmarks were always placed in the same location as used during novel exposure.

### Compounds

The D1/D5 receptor antagonist, SCH23390 (Tocris, Bristol, UK), and the D2/D3 receptor antagonist remoxipride (Tocris, Ellisville, MO, USA) were dissolved in filtered, double distilled water. Compound injections were given as 5 μl, delivered gradually over a 5 min period via a Hamilton syringe (Hamilton Company, Reno, NV, USA). All injections were carried out 30 min prior to stimulation and 30 min *after* recording 30 min of basal synaptic responses (baseline) that were evoked using test-pulse stimulation.

### Data Analysis

**Figure 1:**
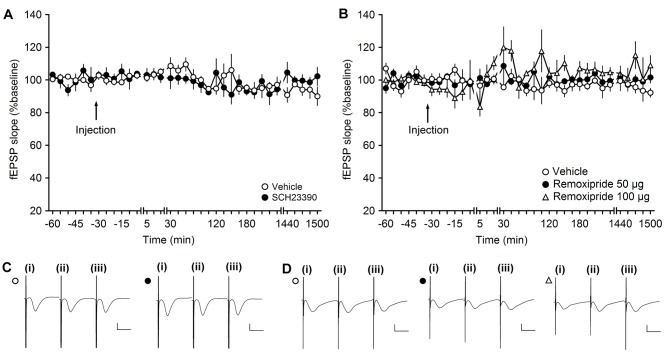
**The D1/D5 receptor antagonist SCH23390 and the D2/D3-receptor antagonist remoxipride have no effect on basal synaptic transmission. (A,B)** In vehicle-injected animals, test-pulse stimulation evoked stable field excitatory postsynaptic potential (fEPSP) responses for the duration of the monitoring period. Injection of the D1/D5 receptor antagonist **(A)** or the D2/D3-receptor antagonist remoxipride **(B)** had no effect on evoked responses. Line breaks indicate change in time scale. **(C)** Analog responses were obtained during a control experiment (open circle) and during an SCH23390 experiment (filled circle); (i) pre-injection; (ii) post-injection; and (iii) 24 h post-injection. Vertical scale bar: 1 mV, horizontal scale bar: 10 ms. **(D)** Analogs depict traces recorded from animal in control experiments (open circle), during an experiment with 50 μg Remoxipride (filled circle) and 100 μg Remoxipride (open triangle); (i) pre-injection; (ii) post-injection; and (iii) 24 h post-injection. Vertical scale bar: 1 mV, horizontal scale bar: 10 ms.

For each time-point, five consecutively evoked responses (evoked at 0.025 Hz) were averaged. The first 30 min of recording (six time-points) served as a reference baseline, and the response obtained at each time-point was expressed as the mean percentage ± the standard error of the mean (SEM) of the reference baseline. Recordings were made every 5 min until 30 min after LFS/HFS and then every 15 min until 4 h had elapsed. The following day an additional 1 h of recordings was obtained. Where the effect of the compounds on basal synaptic transmission was tested, no plasticity-inducing protocol was applied, but the format and duration of the experiment remained the same (see Figure 1). For analysis of differences between groups, multifactorial analysis of variance (ANOVA) with repeated measures was applied. In case, the ANOVA revealed a significant difference, interaction effects at the levels of “time” and “group” were assessed. The level of significance were set at *p* < 0.05.

## Results

### Basal Synaptic Transmission is Not Influenced by Antagonism of D1/D5 or D2/D3 Receptors at MF-CA3 Synapses *In Vivo*

In this study, we tested the influence of D1/D5 or D2/D3 receptor antagonism on synaptic responses in the CA3 region of the hippocampus. To exclude that the application of the antagonists mediated synaptic plasticity changes simply by altering neuronal or synaptic excitability, we first assessed if the doses of antagonists elicited any direct effects on basal synaptic transmission. The doses of antagonists were chosen on the basis of previous experience: the D1/D5 antagonist (SCH23390) dose we used (30 μg) does not affect basal synaptic transmission in the DG, although it does alter persistent synaptic plasticity there (Kulla and Manahan-Vaughan, 2000). The D2/D3 antagonist (remoxipride) dose (50 μg) that we initially tested was previously found to have no effect on basal synaptic transmission in the DG, although it effectively prevented the depressant effects on baseline elicited by D2/D3 agonists (Manahan-Vaughan and Kulla, 2003).

Here, when we applied the D1/D5 receptor antagonist, SCH23390, at a dose of 30 μg (*n* = 4) and recorded test-pulse evoked responses, we detected no significant change in basal synaptic transmission compared to vehicle-treated animals (ANOVA: *F*_(1,6)_ = 0.03, *p* = 0.88; interaction effect: *F*_(22,132)_ = 1.32, *p* = 0.17; *n* = 4). Baseline remained stable for the duration of the 25 h monitoring period (Figures 1A,C).

To test if the D2 receptor antagonist, remoxipride, influences basal synaptic transmission, we first tested the abovementioned dose of 50 μg (*n* = 5). Here, no effect on basal synaptic transmission was observed compared to vehicle-injected animals (*n* = 5; ANOVA: *F*_(1,8)_ = 1.48; *p* = 0.26; interaction effect: *F*_(22,176)_ = 0.58; *p* = 0.93; *n* = 5; Figures 1B,D).

We also tested the higher remoxipride dose of 100 μg (*n* = 5; Figures 1B,D). Here also, no significant effect on basal synaptic transmission was observed (ANOVA: *F*_(1,8)_ = 5.25; *p* = 0.05) although the variability of the evoked responses appeared to increase somewhat. Following application with either of the two doses, basal synaptic transmission remained stable for the 25 h monitoring period.

### Antagonism of D2/D3 Receptors Neither Alters LTP, nor LTD at MF-CA3 Synapses *In Vivo*

At DG synapses, activation of D2/D3 receptors results in a dose-dependent depression of basal synaptic transmission (Manahan-Vaughan and Kulla, 2003). A dose of the D2/D3 receptor antagonist, remoxipride, that completely prevents this agonist-mediated depression has no impact on the profile of persistent (>24 h) LTP that is elicited at these synapses (Manahan-Vaughan and Kulla, 2003). We examined whether the same dose of Remoxipride (50 μg) affects (>24 h) LTP and LTD at MF synapses.

**Figure 2:**
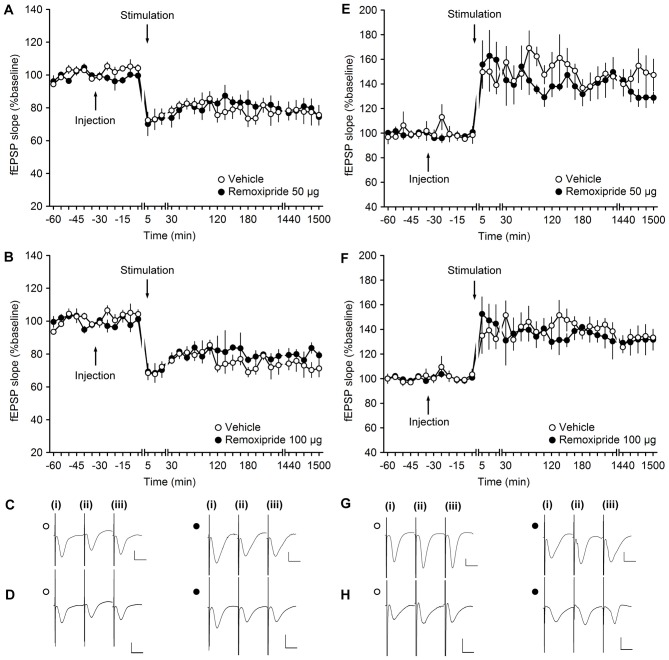
**The D2/D3-receptor antagonist remoxipride does not affect long-term plasticity at mossy fiber (MF)-CA3 synapses. (A,B)** Vehicle-treated animals that received low-frequency stimulation (LFS, 1 Hz, 900 pulses) showed robust long-term depression (LTD) that lasted for over 25 h. LFS in the presence of remoxipride in the doses of 50 μg **(A)** or 100 μg **(B)** expressed long-term potentiation (LTP) that was not significantly different from controls. **(C,D)** Analogs from an LTD experiment that show responses recorded in vehicle-treated animals (open circle) and in animal treated with Remoxipride at a dose of 50 μg **(C)** or 100 μg (**D**; filled circle); (i) pre-LFS; (ii) post-LFS; and (iii) 24 h post-LFS. **(E,F)** High-frequency stimulation (HFS, 4 × 100 pulses at 100 Hz) resulted in robust LTP in vehicle-treated controls, that was unchanged by treatment of the animals with remoxipride in the doses of 50 μg **(E)** or 100 μg **(F)**. Line breaks indicate change in time scale. **(G,H)** Analogs from an LTP experiment that show responses recorded in vehicle-treated animals (open circle) and in animal treated with remoxipride at a dose of 50 μg **(G)** or 100 μg (**H**; filled circle); (i) pre-HFS; (ii) post-HFS; and (iii) 24 h post-HFS. Vertical scale bar: 1 mV, horizontal scale bar: 10 ms.

LFS (1 Hz, 900 pulses) resulted in LTD that lasted for over 24 h in vehicle-treated animals (*n* = 5; Figures 2A,C). LFS in the presence of Remoxipride (50 μg) had no effect on the profile of LTD (ANOVA: *F*_(1,8)_ = 0.06; *p* = 0.81; interaction effect: *F*_(22,176)_ = 0.84; *p* = 0.68; *n* = 5). To be certain that this was not related to the dose of antagonist used, we then applied LFS in the presence of the higher dose of 100 μg of remoxipride (Figures 2B,D). Here, too, remoxipride failed to have any effect on the LTD induced (ANOVA: *F*_(1,6)_ = 1.26; *p* = 0.31; interaction effect: *F*_(22,132)_ = 0.59; *p* = 0.92; *n* = 4).

We then tested the impact of D1/D5 antagonism on LTP. In vehicle-treated animals, HFS (4 × 100 pulses at 100 Hz) resulted in LTP that lasted for over 24 h (Figures 2E,G). Injection of remoxipride (50 μg, *n* = 4) did not affect the profile of LTP compared to vehicle-injected animals (Figures 2E,G). (ANOVA: *F*_(1,6)_ = 0.27; *p* = 0.63; interaction effect: *F*_(22,132)_ = 0.92; *p* = 0.57; *n* = 4). Raising the dose of remoxipride to 100 μg (*n* = 5) also had no impact on LTP (Figures 2F,H; ANOVA: *F*_(1,8)_ = 0.12; *p* = 0.74; interaction effect: *F*_(22,176)_ = 0.81; *p* = 0.72).

### Antagonism of D1/D5-Receptors Inhibits Both Persistent (>24 h) LTP and LTD at MF-CA3 Synapses

**Figure 3:**
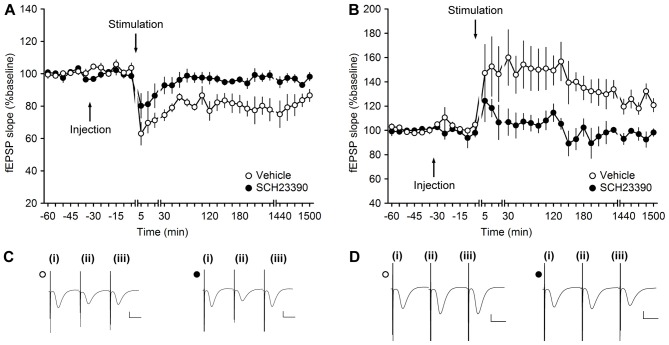
**Antagonism of D1/D5-receptors inhibits synaptic plasticity at MF-CA3 synapses. (A)** LFS (1 Hz, 900 pulses) results in LTD in vehicle-treated animals. LFS in the presence of SCH23390 (30 μg) significantly prevents LTD at MF-CA3 synapses. **(B)** HFS (four trains of 100 pulses at 100 Hz) elicits LTP at MF-CA3 synapses of vehicle-treated animals, whereas antagonism of D1/D5 receptors (SCH23390, 30 μg) significantly prevents LTP. Line breaks indicate change in time scale. **(C)** Analog traces depict fEPSPs recorded at MF-CA3 synapses during an LTD experiment in control animals (open circle) and SCH23390-treated animals (filled circle); (i) pre-LFS; (ii) post-LFS; and (iii) 24 h post-LFS. Vertical scale bar: 1 mV, horizontal scale bar: 10 ms. **(D)** Analog traces depict fEPSPs recorded at MF-CA3 synapses during an LTP experiment; (i) pre-HFS; (ii) post-HFS; and (iii) 24 h post-HFS in the presence of vehicle (open circle) or SCH23390 (filled circle). Vertical scale bar: 1 mV, horizontal scale bar: 10 ms.

Using a dose of the D1/D5 antagonist, SCH23390, that successfully blocks persistent (>24 h) LTP and LTD at DG synapses, we now explored whether persistent synaptic plasticity at MF-CA3 synapses depends on activation of D1/D5 receptors. Here, we observed that SCH23390 (30 μg, *n* = 5), significantly blocked LTD compared to vehicle-treated controls (*n* = 5; Figures 3A,C; ANOVA, *F*_(1,8)_ = 10.70, *p* < 0.05; interaction effect: *F*_(22,176)_ = 0.51, *p* = 0.97).

We then tested the effects of the same dose of antagonist on persistent (>24 h) LTP (*n* = 5; Figures 3B,D). Here, also a significant inhibition of LTP was observed compared to responses evoked in vehicle-treated controls (*n* = 5; ANOVA, *F*_(1,8)_ = 8.99, *p* < 0.05; interaction effect: *F*_(22,176)_ = 0.64, *p* = 0.89).

### Antagonism of D1/D5 Receptors Impairs Learning-Facilitated LTD at MF-CA3 Synapses

Having observed that persistent (>24 h) synaptic plasticity that is induced by patterned stimulation of MF-CA3 synapses, depends on activation of D1/D5 receptors, we went on to explore whether synaptic plasticity that is enhanced by novel spatial learning, also depends on these receptors.

We used an experimental protocol that we have used previously (Hagena and Manahan-Vaughan, 2011). Here, a wHFS protocol (that suffices to induce a transient STP) is applied in conjunction with the exposure of the animal to a novel holeboard. The combination of these two manipulations results in the expression of very robust (>24 h) LTP that is absolutely tied to this novel spatial experience (Hagena and Manahan-Vaughan, 2011). To induce learning-facilitated LTD, a wLFS protocol (that suffices to induce a transient STD) is applied at the same time as the animals explore novel constellations of large (landmark) features that have been introduced into their environment. This results in persistent (>24 h) LTD, that is also directly associated with the learning event (Hagena and Manahan-Vaughan, 2011).

**Figure 4:**
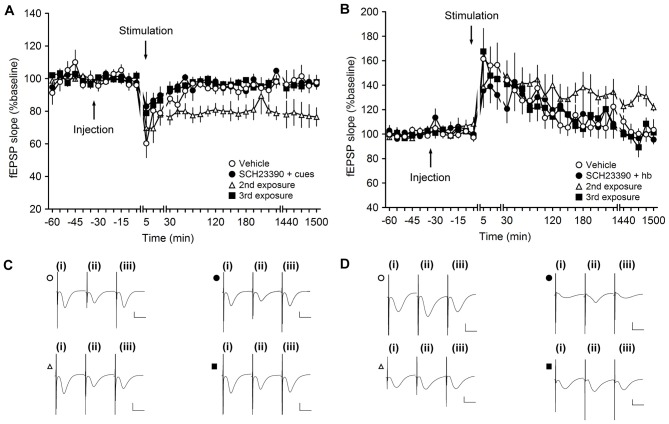
**Antagonism of D1/D5 receptors inhibits learning-facilitated plasticity at MF-CA3 synapses. (A)** Weak low-frequency stimulation (wLFS; 1 Hz, 600 pulses) results in short-term depression (STD) in vehicle-treated animals. Upon first exposure to landmark cues, application of wLFS also resulted in STD in animals that were treated with SCH23390 (30 μg). A second exposure (re-exposure) to the same object-place constellations 1 week later resulted in LTD. When the animals explored the cues for a third time (a further week later), wLFS failed to result in LTD. **(B)** Weak high-frequency stimulation (wHFS, two trains of 100 pulses given at 100 Hz) results in STP in vehicle-treated animals. The first (novel) exposure of the animals to an empty holeboard results in short-term potentiation (STP) in animals that received SCH23390 (30 μg). A second exposure to the same holeboard 1 week later, leads to a facilitation of LTP that lasts for over 24 h. wHFS given during a third exposure to the same holeboard results in STP. Line breaks indicate change in time scale. **(C)** Analogs represent fEPSP responses obtained in an LTD experiment where a vehicle-treated animal received wLFS only (open circle), and Schaffer collateral (SCH)-treated animal explored the novel cues for the first time (filled circle), an animal that we exposed to the cues for a 2nd time (open triangle) or a 3rd time (filled square). The following time-points are shown: (i) pre-wLFS; (ii) post-wLFS; and (iii) 24 h post-wLFS. **(D)** Analogs represent fEPSP responses obtained in an LTP experiment where a vehicle-treated animal received wHFS only (open circle), and SCH-treated animal explored the novel cues for the first time (filled circle), an animal that we exposed to the cues for a 2nd time (open triangle) or a 3rd time (filled square). The following time-points are shown: (i) pre-wHFS; (ii) post-wHFS; and (iii) 24 h post-wHFS. Vertical scale bar: 1 mV, horizontal scale bar: 10 ms.

Weak LFS resulted in STD that was prolonged into LTD that lasts for over 24 h when wLFS was given at the same time as novel exploration of three large objects (landmarks, not shown). When SCH23390 (30 μg, *n* = 4) was applied prior to this protocol, wLFS in conjunction with novel landmark exploration failed to result in an enhancement of STD (Figures 4A,C). The resultant STD was not significantly different from STD that was induced by wLFS in the absence of any behavioral or pharmacological manipulation (ANOVA, *F*_(1,6)_ = 1.73, *p* = 0.24; interaction effect: *F*_(22,132)_ = 1.6, *p* = 0.06; Figures 4A,C).

One week after wLFS/landmark exposure in the presence of SCH23390, we exposed the animals to the same object-place constellations anew (2nd exposure). The landmarks were returned to their original positions. Here, we observed that wLFS now succeeded in facilitating STD into LTD (Figures 4A,C; ANOVA, *F*_(1,6)_ = 9.4, *p* < 0.05; interaction effect: *F*_(22,132)_ = 0.79, *p* = 0.74), in line with the likelihood that SCH23390 during the original object place exposure had prevented learning of this experience (Lemon and Manahan-Vaughan, 2006).

This finding aligns with previous reports that show that preventing learning-facilitated plasticity prevents memory of the object-place experience (Popkirov and Manahan-Vaughan, 2011). The second exposure of the animals to the objects was, thus, perceived by the animals as being a novel experience. If this is the case, one would expect that a further exposure of the animals to the same object-place constellations (a further 1 week later/3rd exposure) would fail to facilitate LTD, as the animals have now learned and remember the object-place constellations. This was indeed the case: wLFS during a third object-place exposure did not result in persistent LTD (Figures 4A,C; ANOVA, *F*_(1,6)_ = 0.03, *p* = 0.87; interaction effect: *F*_(22,132)_ = 0.83, *p* = 0.69).

### Antagonism of D1/D5 Receptors Impairs Learning-Facilitated LTP at MF-CA3 Synapses

We then tested the D1/D5 receptor-dependency of LTP that is typically facilitated by novel spatial learning. In this paradigm, it is the exposure of the animals to a novel spatial change such as the introduction of a holeboard to the recording chamber that brings about the strengthening of STP into LTP (Hagena and Manahan-Vaughan, 2011).

Weak HFS resulted in STD in vehicle-treated animals (*n* = 6; Figures 4B,D). Coupling of wHFS with the exploration of a novel holeboard, in the presence of SCH23390 (30 μg, *n* = 6), failed to produce the characteristic facilitation of STP into LTP (ANOVA, *F*_(1,10)_ = 0.03, *p* = 0.86; interaction effect: *F*_(22,220)_ = 1.20, *p* = 0.25). As mentioned above, previous studies have shown that antagonism of D1/D5 receptors prevents spatial learning (Lemon and Manahan-Vaughan, 2006). Thus, re-exposure of the animals to the same holeboard (in the absence of the antagonist) should be perceived as a novel spatial learning event. When the animals were re-exposed to the holeboard 1 week after the first event (2nd exposure) application of wHFS in conjunction with holeboard exposure resulted in robust LTP that lasted for over 24 h (Figures 4B,D). Effects were significantly different from 4 h onwards (ANOVA_T17–25_, *F*_(1,10)_ = 6.04, *p* < 0.05; interaction effect: *F*_(10,100)_ = 0.65, *p* = 0.77). Following the same logic as described for the LTD experiment (Figures 4A,C), a 3rd exposure to the same holeboard would be expected to fail to strengthen synaptic potentiation. This was indeed the case: under these circumstances wHFS in conjunction with holeboard exposure resulted in STP that was not significantly different from animals that received wHFS in the absence of any behavioral or pharmacological manipulation (ANOVA, *F*_(1,10)_ = 0.002, *p* = 0.96; interaction effect: *F*_(22,220)_ = 0.66, *p* = 0.88), in line with previous reports that it is the novelty of the holeboard, and its association with the learning event, that facilitates STP in LTP (Kemp and Manahan-Vaughan, 2007).

## Discussion

This study addressed the previously uncharted role of the dopaminergic system in the neuromodulation of very persistent forms of synaptic plasticity at MF-CA3 synapses of freely behaving rats. We report that synaptic plasticity at these synapses does not depend on activation of D2/D3-receptors. By contrast, synaptic plasticity that is either induced by patterned stimulation of MFs, or by coupling subthreshold afferent activation with a novel spatial learning event, critically depends on the activation of D1/D5 receptors. Both synaptic plasticity and learning/memory are highly dependent on the degree of activation of neuromodulators. Previous studies have shown that neuromodulators, such as serotonin or noradrenaline, exert a very tight regulatory control over synaptic plasticity processes at MF-CA3 synapses of behaving rats (Hagena and Manahan-Vaughan, 2012; Twarkowski et al., 2016). Here, we report that DA, acting on D1/D5 receptors also potently modulates MF-CA3 plasticity *in vivo*.

By contrast, D2/D3 receptors do not appear to play an important role in persistent forms of synaptic plasticity at MF-CA3 synapses: we observed that antagonism of D2/D3 receptors had no impact on LTP or LTD at these synapses. This is in line with other reports as to the rather peripheral role of this receptor for hippocampal synaptic plasticity (Manahan-Vaughan and Kulla, 2003). Although antagonism of this receptor had no effect on persistent LTP in the DG of behaving rats, it was reported that receptor antagonism prevents depotentiation (Manahan-Vaughan and Kulla, 2003). This suggests that D2/D3 receptors may contribute more subtly to synaptic plasticity, whereby activation of these receptors may serve to curtail LTP. These properties may be state-dependent: studies performed on rat hippocampal slices showed that pharmacological activation of D2 receptors can prevent LTD in the CA1 region (Chen et al., 1996), whereas agonist activation of D2/D3 receptors elicits a dose-dependent decrease in synaptic transmission in the DG *in vivo* (Manahan-Vaughan and Kulla, 2003). In other words, it may be the relative activation of D2/D3 receptors (under naturalistic conditions), as well as their relative location within the hippocampus that determines their direct contribution (or lack of it) to synaptic plasticity. The expression level of D2 receptors in the CA3 region is relatively sparse, and has been reported as being non-existent at MF-CA3 synapses (Goldsmith and Joyce, 1994; Gangarossa et al., 2012). This is one possible explanation for the lack of effect of D2/D3 antagonism on synaptic plasticity at MF-CA3 synapses. Interestingly, hyperdopaminergia, whereby excessive synaptic levels of DA chronically occur, is associated with impaired CA1 LTD (Morice et al., 2007). Effects are prevented by a DA D2 antagonist (Morice et al., 2007), suggesting that the D2 agonist-mediated prevention of LTD, described in the Chen et al. (1996) *in vitro* study, may have emulated a pathological, rather than a naturalistic state of activation of D2 receptors in the hippocampus.

The absence of impact of D2/D3-receptor antagonism on persistent synaptic plasticity in behaving rats, as seen in our study, is also in line with reports of only a minor contribution of D2-receptors in fear memory consolidation in the CA3 region (Wen et al., 2014). Descriptions of a contribution of D2/D3 receptors to hippocampus-dependent learning may thus derive from actions of DA on D2/D3 receptors in hippocampal subfields other than the CA3 region (Goldsmith and Joyce, 1994; Gangarossa et al., 2012), or on different components of the longitudinal axis of the hippocampus (Chen et al., 1996; Wilkerson and Levin, 1999; Manahan-Vaughan and Kulla, 2003; Stuchlik et al., 2007). In the *in vivo* studies of the DG, strong agonist activation of D2/D5 receptors leads to reductions in basal synaptic transmission (Kulla and Manahan-Vaughan, 2000). Learning deficits that are associated with an overactivation of D2 receptors (Morice et al., 2007) may thus, relate to D2/D5 receptor-mediated suppression of hippocampal information processing.

In contrast to the lack of effect of D2/D5 receptor antagonism on MF-CA3 LTP and LTD, we observed significant impairments of both forms of plasticity when D1/D5 receptors were antagonized. We observed that not only synaptic plasticity that is induced solely by patterned afferent stimulation, but also synaptic plasticity that is facilitated by novel learning, depends on D1/D5 receptor activation at MF-CA3 synapses. In hippocampal slices, DA potentiates MF-CA3 synaptic transmission by acting on presynaptic D1-like receptors (Kobayashi and Suzuki, 2007) and in the CA1 region *in vitro*, supports spike-timing dependent plasticity also via activation of D1 receptors (Edelmann and Lessmann, 2013). In the former study, a specific dopaminergic modulation of both AMPA and NMDA receptor responses was reported at MF synapses, which offers a possible explanation as to why not only the late protein synthesis-dependent components of LTP/LTD (Hagena and Manahan-Vaughan, 2013) but also the early components of these forms of plasticity were affected by D1/D5 receptor antagonism in the present study. That said, MF-CA3 synaptic plasticity does not depend on NMDA receptor activation *in vitro* or *in vivo* (Harris and Cotman, 1986; Hagena and Manahan-Vaughan, 2010). Very rapid effects of protein translation inhibitors on persistent forms of LTP and LTD have been reported at MF-CA3 synapses *in vivo*, however (Barea-Rodríguez et al., 2000; Hagena and Manahan-Vaughan, 2013). Activation of D1 and D5 receptors induces a signaling cascade that leads to the activation of cyclic 3′,5′-monophosphate (cAMP; Missale et al., 1998; Vallone et al., 2000; Undieh, 2010), and long-term forms of synaptic plasticity depend on the activation of cAMP at MF-CA3 synapses (Huang et al., 1994; Weisskopf et al., 1994). Antagonizing D1 receptors suppresses the expression of the later phases of CA1 LTP *in vitro* (Frey et al., 1991; Huang and Kandel, 1995) and activation of D1/D5 receptors induces a protein synthesis-dependent late potentiation in the CA1 region (Huang and Kandel, 1995). Negative modulation of cAMP rapidly induces LTD in MF-CA3 synapses (Tzounopoulos et al., 1998). Thus, the effects of the D1/D5 antagonist on both the early and late phases of plasticity observed in our study may relate to D1/D5 receptor modulation of cAMP levels and the resultant modulation of downstream signaling cascades.

The impairment by D1/D5 receptor antagonism of persistent LTP and LTD at MF-CA3 synapses has interesting implications for hippocampus-dependent information processing and encoding. MF-CA3 synapses encode information about novel spatial experience by means of LTP and LTD (Hagena and Manahan-Vaughan, 2011) and the CA3 region is proposed to comprise a locus for pattern completion and/or pattern separation (Kesner and Warthen, 2010; Neunuebel and Knierim, 2014; Lee et al., 2015; Knierim and Neunuebel, 2016). Transgenic mice that lack the D1 receptor exhibit impaired spatial learning, fear learning and classical conditioning responses (Ortiz et al., 2010). Context-dependent extinction learning (André and Manahan-Vaughan, 2016), contextual aversive learning (Broussard et al., 2016), object-place learning (Lemon and Manahan-Vaughan, 2006, 2012) and learning-facilitated synaptic plasticity at other hippocampal synapses such as the Schaffer collateral-CA1 synapse (Lemon and Manahan-Vaughan, 2012) and the PP-DG synapse (Wiescholleck and Manahan-Vaughan, 2014) are also all modulated by D1/D5 receptors. This multi-faceted regulation of diverse forms of hippocampus-dependent learning, as well as distinct forms of persistent synaptic plasticity in all three hippocampal subfields indicates that DA acting on D1/D5 receptors enables a potent and significant neuromodulation of hippocampal function. This is likely to support the known roles of the dopaminergic system in reward (Baudonnat et al., 2013) and punishment-based learning (Carlezon and Thomas, 2009), as well as attention and motivation (Hansen and Manahan-Vaughan, 2014).

## Conclusion

D1/D5 receptor activation is crucially involved in long-term synaptic plasticity in various regions of the hippocampus. This in turn is likely to support dopaminergic regulation of lasting memory. Here, we show that D1/D5 but not D2/D3 receptors contribute to very persistent forms of synaptic plasticity that are either elicited solely by patterned afferent stimulation, or by coupling afferent stimulation with novel spatial learning.

Activation of these receptors under specific circumstances, such as novelty or arousal, triggers the release of DA that activates D1/D5 receptors and supports the encoding of experience. The activation of D1/D5 receptors at MF-CA3 synapses may serve to support MF-specific memory and encoding functions, such as pattern separation, or an increased effectivity in working memory tasks. Taken together, our findings support that DA acting on D1/D5 receptors plays a decisive role in specific (long-lasting) forms of memory encoding in the hippocampus.

## Author Contributions

DM-V and HH created study concept. HH conducted the experiments, prepared the figures and data overviews. HH and DM-V performed the data analysis. DM-V wrote the article with contributions from HH.

## Conflict of Interest Statement

The authors declare that the research was conducted in the absence of any commercial or financial relationships that could be construed as a potential conflict of interest.
